# From kitchen to clinic: cherry tomato model for sub‐Tenon's block training

**DOI:** 10.1002/anr3.12321

**Published:** 2024-09-05

**Authors:** F. Lersch, T. Schweizer, J. M. Berger‐Estilita

**Affiliations:** ^1^ Department of Anaesthesiology and Pain Medicine Inselspital, Bern University Hospital, University of Bern Bern Switzerland; ^2^ Department of Anaesthesiology and Pain Medicine Inselspital, Bern University Hospital, University of Bern Bern Switzerland; ^3^ Institute of Anaesthesiology and Intensive Care Salem Spital, Hirslanden Hospital Group Bern Switzerland; ^4^ Institute for Medical Education University of Bern Bern Switzerland; ^5^ CINTESIS@RISE, Centre for Health Technology and Services Research, Faculty of Medicine University of Porto Porto Portugal

**Keywords:** anaesthesia, Tenon capsule, education, medical, simulation training

**Figure 1:**
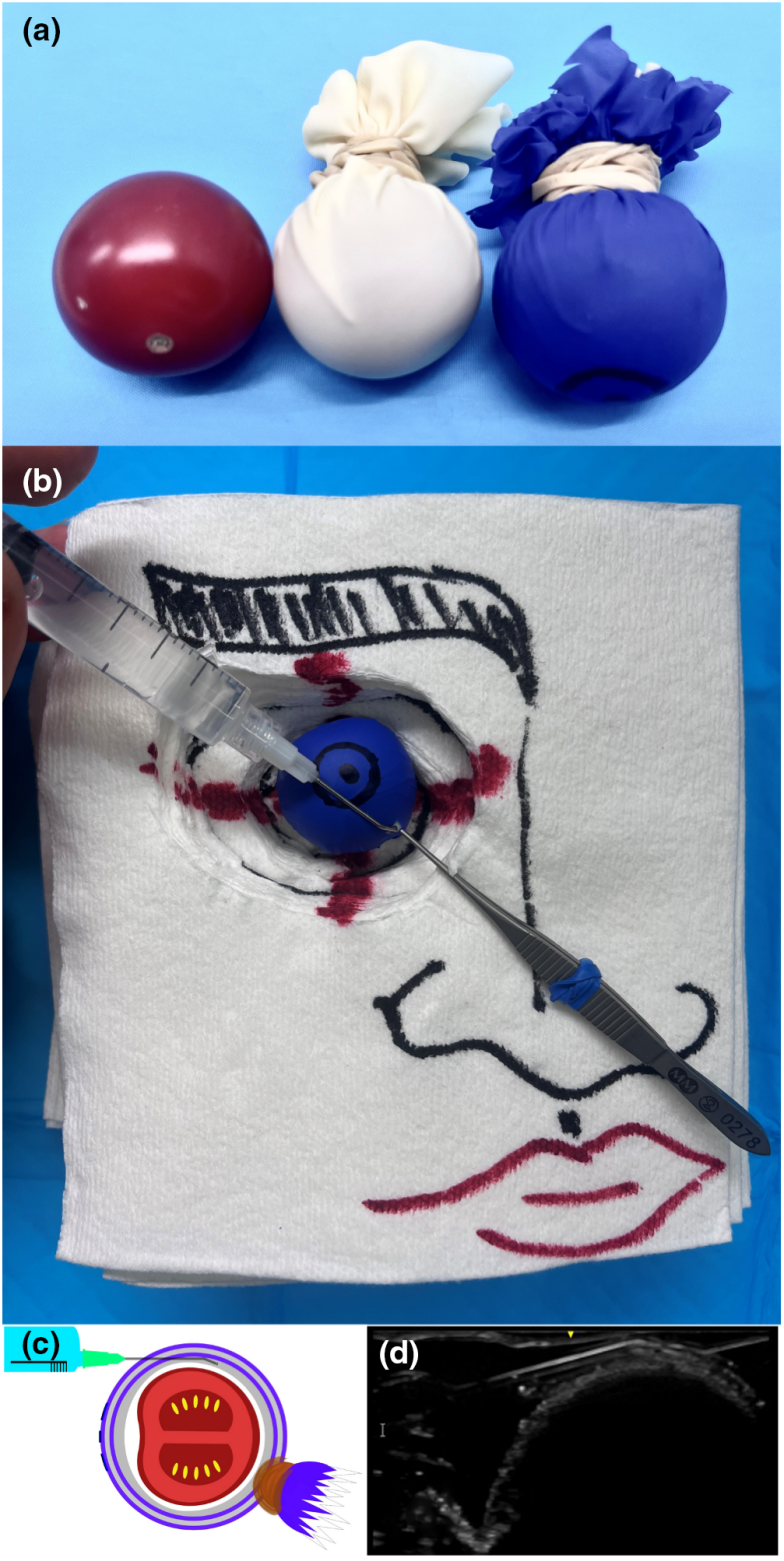
Construction of the simulated eye (a): The cherry tomato (left) is wrapped inside a white rubber glove representing the sclera (centre), then in a double layer of coloured glove representing the bulbar conjunctiva and Tenon's fascia (right) on which the pupil and limbus are drawn. A paper towel makeshift model of a face provides an orbit in which the cherry tomato‐model is placed (b). The red cross represents the rectus muscles and the four quadrants. Instruments shown are positioned to maximise distance from rectus muscles in the inferonasal quadrant. Graphic caption of the model (c) alongside ultrasound image of angulated sub‐Tenon's block cannula in use on the model (d). Applying small quantities of ultrasound jelly (c; grey line) between the rubber layers helps locate the correct space with the cannula and facilitates ultrasound examination.

Our centre uses a cherry tomato model to simulate the anatomical structures of the vitreous body and surrounding tissues in training for sub‐Tenon block administration [1]. This model provides a hands‐on, anatomically accurate simulation that allows trainees to practice and refine their skills under the guidance of experienced instructors. It eliminates the need for training teams to use animal cadaver eyes [2]. We use a cherry tomato to simulate the vitreous body, surrounded by rubber gloves representing the tissue layers involved in sub‐Tenon block administration. A cherry tomato is wrapped in a white rubber glove, simulating the sclera, and then a double layer of coloured gloves simulating the bulbar conjunctiva and Tenon's capsule (Fig. 1a). The pupil and limbus are marked or glued on the outer glove layer helping trainees judge the distance to the incision. The spherical cherry tomato simulates the vitreous body of the eye, allowing trainees to practice manoeuvring around a similarly sized and shaped object. The importance of the coloured double layer (conjunctiva and Tenon's capsule) is stressed in practical training as both layers must be engaged and lifted off the sclera before opening the potential space between the Tenon's capsule and the sclera. Having the contrasting white layer (sclera) appear during practice is essential, as is gliding the cannula behind the eye on the sclera. Identification of the plane and the gliding sensation can be enhanced by positioning a layer of ultrasound jelly between the simulated sclera and the Tenon's capsule (Fig. 1c; grey line). This also enables an ultrasound examination of the model and unequivocally demonstrates the layers (Fig. 1d). Supplementary videos S1 and S2 show the construction and use of the model, respectively.

The training program includes pre‐instructional videos and literature (Table 1). Trainees receive instruction during dedicated time without interruptions [3]. The training involves an explanation of the eye quadrants and the necessity of maintaining a safe distance from the eye muscles. Trainees receive instruction on using forceps and scissors to breach the conjunctiva‐Tenon's capsule double layer, ensuring the secure placement of a blunt cannula on the sclera. Instructors also demonstrate the double layer using ultrasound (Fig. 1d). Trainees are encouraged to perform at least five sub‐Tenon's blocks on the model using the inferonasal quadrant. Instructors emphasise the layers in the model, provide feedback on the correct use of instruments and emphasise the importance of slowly injecting 2–5 ml of local anaesthetic. By integrating the cherry tomato model into a training package, trainees gain theoretical knowledge and practical skills in sub‐Tenon's administration [4]. Overall, this package provides hands‐on, anatomically accurate simulation [5] which allows trainees to practice and refine their sub‐Tenon administration skills under experienced instructors' guidance.

**Table 1:** Links to videos of the simulated procedure trainees are required to watch before training on the model.

**Link 1:** https://vimeo.com/390905369 (9 min 43 s)
**Link 2:** https://www.roah.ch/2019/10/14/teaching‐video‐sub‐tenon‐peribulbar‐blocks/ (8 min 05 s)
**Link 3:** https://www.roah.ch/2020/05/08/sub‐tenons‐block‐training‐model‐how‐to‐put‐cherry‐tomatoes‐to‐good‐use/ (3 min 25 s)
